# College and Hospital Notes

**Published:** 1898-11

**Authors:** 


### COLLEGE AND HOSPITAL NOTES.

The Samaritan Hospital, at Troy, N. Y., was recently opened and is in every way a model of its class. It contains eighty ward beds, thirty private rooms and an infectious building in four separate departments, containing thirty-five beds. Dr. William Wotkyns Seymour is attending surgeon.

---

The following appointments were recently announced at the Buffalo Hospital of the Sisters of Charity: Dr. John H. Pryor, consulting physician; Dr. Stephen Y. Howell, gynecologist; Dr. Eugene A. Smith, surgeon; Dr. H. Y. Grant, ophthalmologist and otologist.

CORNELL University Medical College, recently established in New York City, has received a princely gift from Col. Oliver H. Payne, which includes a valuable site in First Avenue, money for the erection of buildings and an endowment fund—the whole aggregating the munificent sum of $1,500,000.

